# Assessment of psychometric properties of the Persian version of the spiritual care competency self-assessment tool

**DOI:** 10.1017/S147895152400141X

**Published:** 2025-01-21

**Authors:** Amir Jalali, Fatemeh Merati Fashi, Mohammad Karami, Parnia Kalhory, Nazanin Mardani Taghvostani, Khalil Moradi

**Affiliations:** 1 Substance Abuse Prevention Research Center, Research Institute for Health, Kermanshah University of Medical Sciences, Kermanshah, Iran; 2 Department of Emergency and Critical Care Nursing, School of Nursing and Midwifery Kermanshah University of Medical Sciences, Kermanshah, Iran; 3 Department of Islamic Education, School of Medicine Kermanshah, University of Medical Sciences, Kermanshah, Iran; 4 Department of Nursing, Student Research Committee, Kermanshah University of Medical Sciences, Kermanshah, Iran

**Keywords:** Validity, reliability, nursing and midwifery, spiritual care, competency

## Abstract

**Background:**

Spiritual care is essential for the health and well-being of patients and their families, so nursing and midwifery students should have professional competency in this field.

**Objectives:**

The present study aimed to translate the Spiritual Care Competency Self-Assessment Tool for nursing and midwifery students into Persian and evaluate its psychometric properties.

**Methods:**

This study has a methodological study design.

**Methods measures:**

The present study was conducted from July 4 to November 19, 2023, at the Faculty of Nursing and Midwifery in west of Iran. The tool was translated into Persian using the forward-backward translation method. The construct validity was examined using exploratory factor analysis (EFA) and confirmatory factor analysis (CFA) with a total of 536 nursing and midwifery students. The internal consistency was assessed using Cronbach’s alpha coefficient. Also, the reliability of the tool was evaluated using the test–retest method. SPSS version 26 and Lisrel version 8 software were used in this study.

**Results:**

Face and content validity was confirmed quantitatively and qualitatively. The results of EFA and CFA confirmed the tool with 4 factors and 28 items. CFA results indicated a well-fitting model (comparative fit index [CFI] = .97, Non-Normed Fit Index (NNFI) = .92, goodness of fit index [GFI] = .91, root mean square error of approximation [RMSEA] = .05, Standardized Root Mean Square Residual (SRMR) = .046). Pearson’s correlation coefficient confirmed a significant relationship between items, subscales, and the main scale. Also, Cronbach’s alpha coefficient (.968) and test–retest (.867) confirmed the reliability of the Persian version of the tool.

**Conclusion:**

The present study showed that the Persian version of the EPICC Spiritual Care, with 4 factors and 28 items, was suitable for validation and that its psychometric properties were acceptable according to COSMIN criteria. In general, the results showed that the Persian version of the EPICC Spiritual Care is a valid and reliable tool that students, preceptors, and educators can use in clinical settings as a practical way of discussing and evaluating spiritual care competency in Iran.

## Introduction

The International Council of Nursing announced in 2021 that spiritual care is part of the responsibility of nurses and midwives (Nursing 2018). And as a component, it shows the quality of care (Ghorbani et al. 2021) This multidimensional concept is so crucial that the National Health Service Education Center of Scotland defined it as the care nurses provide to meet the spiritual needs of people who face trauma, illness, or grief (Ross et al. 2018). Additionally, the concept of spiritual care has been incorporated in the nursing theories of Newman, Travelbee, and Henderson (Butts and Rich 2021) and documented in the North American Nursing Diagnosis Association (NANDA) and the Nursing Interventions Classification (NIC) as a diagnosis and an intervention (Diagnoses 2018). Nursing care aims to promote health, prevent diseases, maintain health, and reduce pain and discomfort, and spiritual care can significantly contribute to achieving this goal (Babamohamadi et al. 2018).

Nurses and midwives have a positive attitude toward providing spiritual care, but they often face various challenges that prevent them from doing so adequately. These challenges include lack of education, skill, knowledge, time, personal discomfort, and fear (Taylor et al. 2023). According to a systematic review by Lewinson et al. (2015), nurses felt incompetent in providing spiritual care and required more knowledge and skills in this area. This gap and challenge were also observed among undergraduate and graduate students by Lewinson et al. (2015). McSherry et al. (2021b) reported that many nurses and midwives needed a proper understanding of spirituality and related care and more readiness and skill to perform such care (McSherry et al. 2021b).

Furthermore, studies have shown that nurses do not receive adequate education in spiritual care, both during and after their studies (Cetinkaya et al. 2013; Lewinson et al. 2015). As a result, spiritual care is part of the phenomenon of “lost care” (Chaboyer et al. 2021; Parozzi et al. 2023). A European study of 6 universities in 4 countries also suggested that students’ comprehension of spiritual care and personal spirituality were crucial factors affecting their perception of competence in spiritual care. These factors could also strongly predict students’ self-reporting ability to provide spiritual care (Cone and Giske 2018). Therefore, some international studies have recommended various strategies, such as developing spiritual care competence or incorporating spiritual care into curricula (Giske et al. 2023).

In Iran, the topic of spiritual care delivery by health-care professionals, particularly nurses, is gaining heightened significance. However, the educational programs incorporated into Iran’s nursing curriculum do not adequately equip nursing students with the essential skills required for the effective delivery of spiritual care (Sedighie et al. 2021). In Iran, the barriers to providing spiritual care in health-care environments are complex and varied. Nurses face obstacles such as non-compliance with human resource standards, lack of attention of organizational managers to the importance of holistic care, motivational, environmental, and training barriers, as well as barriers to interprofessional collaboration and communication (Momeni et al. 2022). Additionally, one of the primary obstacles to providing spiritual care to patients is the insufficient preparedness of nurses, which stems from a lack of comprehensive and proper training (Sedighie et al. 2021). Ultimately, enhancing the educational curriculum with reformed programs and bolstering organizational support can lead to an improvement in the quality of spiritual care provided to patients in Iran.

Generally, the critical significance of delivering spiritual care within nursing and midwifery disciplines mandates that professionals in these fields maintain a high degree of proficiency to provide such care effectively (Ross et al. 2021). Therefore, evaluating the competencies of nursing and midwifery students in delivering spiritual care is of considerable significance. This evaluation is vital for improving their capacity to provide high-quality spiritual care that is culturally responsive in health-care settings (McSherry et al. 2021a). Furthermore, this evaluation not only elucidates the strengths and weaknesses but also fosters opportunities for advancing spiritual care by comprehending diverse beliefs and augmenting spiritual evaluations (Cone et al. 2023). Hence, the availability of a tool capable of assessing the clinical competencies of nursing and midwifery students holds significant importance.

Some studies have considered students’ self-evaluation to enhance the skill of spiritual care in clinical settings. Using a self-reporting approach, students could gain a deeper and more accurate insight into themselves, internalize learning, and have more realistic expectations of themselves and others (Yousefzadeh Chosary 2017). Although various methods have been used to measure the structure of spiritual care, only some tools are suitable for assessing students’ competence in providing spiritual care. The most widely used tool at the international level is the self-assessment tool for spiritual care competence (EPICC Spiritual Care) in nursing and midwifery students, which Tove Giske et al. designed and psychometrically tested in 2022 (Giske et al. 2023). This self-assessment tool is a competency framework for nurses and midwives based on previous European research. It comprises 4 sub-scales of competence with 5–8 items each (28 items in total). The sub-scales are: Intrapersonal spirituality, Interpersonal spirituality, Assessment and planning of spiritual care, and Intervention and evaluation of spiritual care. This instrument not only offers a concrete approach for evaluating knowledge, skills, and attitudes related to spirituality, but it also has the potential to establish standards for cultivating students’ competencies in the delivery of spiritual care (Giske et al. 2023).

Considering the holistic perspective in nursing, and the complete areas of spiritual care in this scale, which has strong individual interactions and aspects of spirituality, this tool was selected for study. In addition, spiritual care is a comprehensive and complete care that is done according to spirituality and its related dimensions. In this regard, people’s religion and religious inclinations, philosophy of life, and culture are influential in it (Murgia et al. 2022). Many studies have acknowledged that spirituality depends on the context and culture of society (Arrey et al. 2016; Carr 2018; Jensen 2021; Vu and Burton 2022). Therefore, the validated tools that match Iranian society’s cultural and religious background are needed. However, no scale exists for self-assessment of spiritual care competence by nursing and midwifery students in Iran. This study aimed to reveal the differences in the provision of spiritual care by translating and psychometrically testing the EPICC Spiritual Care tool in Iranian nursing and midwifery students.

## Methods

### Study design

This methodological and cross-sectional study was performed from July 4 to November 19, 2023 to determine the psychometric evaluation of the Persian version of the Spiritual Care Competency Self-Assessment Tool among student nurses and midwives, which was executed in 2 main phases: the translation and cultural adaptation phase, followed by the psychometric evaluation phase.

## Participants and setting

The sampling method for examining validity and reliability was proportionate stratified sampling among nursing and midwifery students from 4 the Faculty of Nursing and Midwifery in west of Iran (Kermanshah, Ilam, Kurdistan, Hamadan). A sample of 536 nursing and midwifery students completed the questionnaire.

### Inclusion and exclusion criteria

The inclusion criteria for the study were interest and satisfaction in participating and being a nursing and midwifery student (first year to fourth year student). (Nursing and midwifery students from the second semester onward, these students have learned care topics in the first semester and attended clinical departments.)

If a specific questionnaire was completed less than 90%, it was excluded from the study.

Before completed the questionnaire, we secured verbal consent from every participant. Should any individual have indicated a lack of interest or opted out, we honored their choice and refrained from gathering any data from them.

### The EPICC Spiritual Care Competency Self-Assessment Tool

Giske et al. developed the main scale in 2023, which has 28 items and 4 factors: interpersonal spirituality (7 items), intrapersonal spirituality (5 items), spiritual-care assessment planning (8 items), and spiritual-care intervention evaluation (8 items). The scale is a self-assessment tool that evaluates individuals’ ability to provide spiritual care. The items are presented as Likert scale questions with 5 response options, ranging from “Completely disagree” (1) to” Completely agree” (5). The instrument’s Cronbach’s alpha reliability coefficient is .91, with factor reliabilities reported between .7 and .8 (Giske et al. 2023).

### The translation and cultural adaptation phase

The 10-step method of Wild et al. (2005) was used to perform the cultural validation steps of the tool. In the first step, permission was obtained from the original tool designer. The tool was translated from English into Persian and then back into English using the forward-backward method. Two independent translators were asked to translate the tool from English into Persian at the same time. The research team reviewed and combined the 2 translated versions into a single one. This Persian version was simultaneously and independently back-translated from Persian into English by 2 other translators who were independent of the translators in the first stage. The research team reviewed the 2 resulting English versions and obtained a single version, which was sent to the tool developer, and their recommendations were applied to the final version. The last Persian version was given to 12 nursing and midwifery students, who were asked to identify any ambiguous points and possible errors. The research team reviewed and incorporated the students’ opinions in the final version. A Persian language and literature expert edited and approved the final version. The final version was used for psychometric evaluations (validity and reliability).

### The psychometric evaluation phase

**Figure 1.**
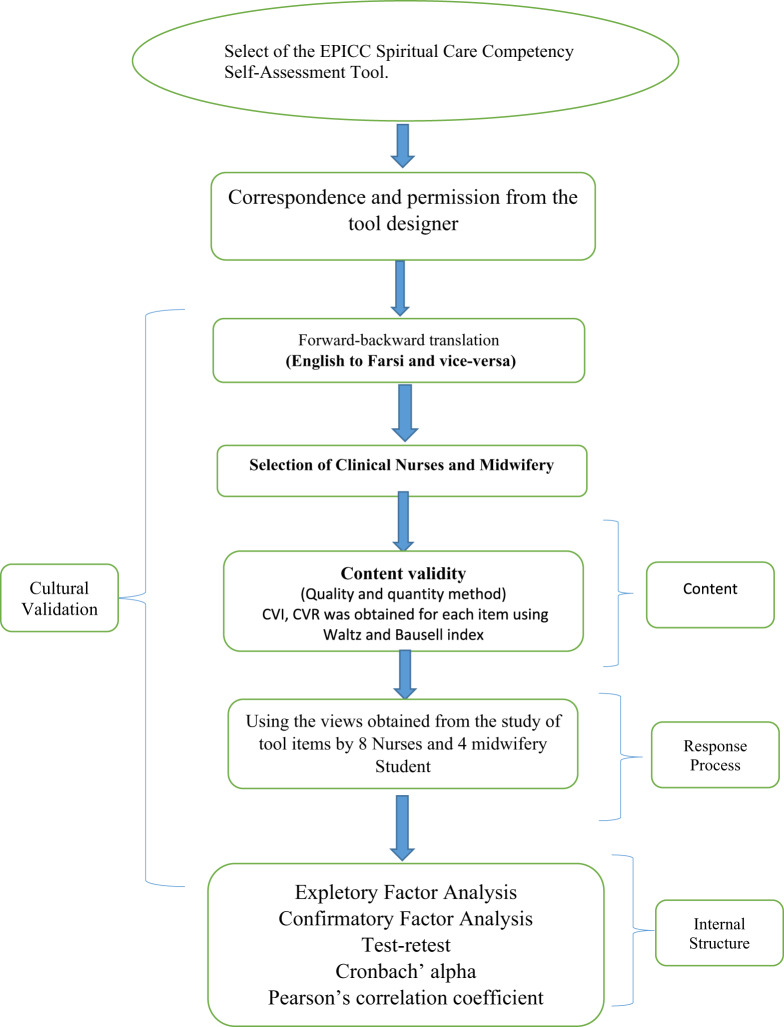
Response process.

In this phase, we performed psychometric evaluations (the face, content, and construct validity, along with the reliability) as detailed in the following sections (Figure 1).

### Face validity assessment (qualitative and quantitative)

In the qualitative phase, the evaluation of the tool items in terms of understandability, absence of ambiguity, and the appropriate relation among them was conducted by a group including 12 nursing and midwifery students who were not part of the initial sample (Taghizadeh et al. 2017). In the quantitative phase, this group of students was asked to rate the significance of each item using a 5-point Likert scale (1 = Not important at all to 5 = Very important). Following the calculation of each item’s impact score, those items with an impact score exceeding 1.5 were selected for retention (Mohammadbeigi and Aligol 2015).

### Content validity assessment

#### Qualitative content validity assessment

In this stage, 14 experts in the field of nursing and instrument development assessed the items of the translated version of the instrument for syntax, phrasing, clarity, and cultural compatibility with the Iranian culture.

#### Quantitative content validity assessment

The content validity ratio (CVR) and the content validity index (CVI) were used to assess the content validity of the instrument. A group consisting of 14 specialists was invited to rate the necessity of the instrument items on a 3-point Likert scale as “Essential,” “Useful but not essential,” and “Unessential” to calculate the CVR. Additionally, their suggestions for revisions on the item wording were collected and incorporated into the final version (Sharif Nia et al. 2020) The Lawshe method was used to calculate the CVR of the instrument based on their rating scores. The minimum acceptable value for the CVR, considering the panel of 14 experts, was determined to be .51 (Lawshe 1975).

The CVI can be used to assess the relevance of the instrument’s items at both the individual item level (I-CVI) and the overall scale level (S-CVI). Therefore, in order to evaluate the relevance of the items, the same 14 experts were asked to rate them using a 4-point Likert scale from 1 to 4. Also, by taking the ratio of experts who gave a relevance rating of 3 or 4 to the overall number of experts, the I-CVI was calculated. Items that registered a CVI value above .79 were deemed suitable, those with CVI values from .70 to .79 required modifications, and items with CVI values below .70 were judged as unsatisfactory and thus excluded (Polit et al. 2007). Also, by averaging the CVI values of all items, the S-CVI was determined. An S-CVI value of .9 or above signifies that the scale being evaluated has good content validity (Polit-O’Hara and Yang 2016).

### Construct validity assessment

A test demonstrates construct validity when the scores derived from its administration correlate with the intended concepts or theoretical constructs (22). For assessing the construct validity of the exploratory factor analysis (EFA) and confirmatory factor analysis (CFA) were employed.

Considering translating the tool into Persian and using it in a different cultural context than the original version, as well as uncovering hidden variables based on Iranian society’s culture, we first conducted an EFA followed by a CFA (Sharif-Nia and Hanifi 2023).

Out of 536 total participants, 200 were chosen for EFA, and the remaining 336 were used for CFA. In this study, EFA was performed utilizing Varimax rotation. The researchers established that eigenvalues should exceed 1 and factor loadings are commonly considered meaningful in exploratory analyses when they exceed 0.4 (Floyd and Widaman 1995; Wu 2010). The Kaiser–Meyer–Olkin (KMO) and Bartlett’s tests were utilized to evaluate the sampling adequacy. KMO values of more than .9 are considered to be excellent, and of more than .8 are regarded as satisfactory, and the value for Bartlett’s test should be below 0.05 (*p* < .05) (Magolda 1987).

In this phase, 200 students participated in the EFA stage. Shrestha demonstrated that a sample size of 200 participants is often considered sufficient as an absolute criterion for conducting EFA (Shrestha 2021).

After the EFA, we conducted a CFA using structural equation modeling to validate the factor structure unearthed in the EFA (Harrington 2009). CFA demonstrates the effectiveness of each item in measuring the various dimensions of the scale. The assessment of the model fit indices relied on the subsequent criteria: The ratio of chi-square to its degree of freedom (*χ*^2^/*df*) < 3, root mean square error of approximation (RMSEA) < .08 (Byrne 2013), goodness of fit index (GFI) > .90, comparative fit index (CFI) > .90, Tucker–Lewis index (TLI) > .90, incremental fit index (IFI) > .90, and adjusted goodness of fit index (AGFI) > .80 (Floyd and Widaman 1995). During this phase, the CFA stage of the instrument was carried out with the participation of 336 students. Generally, it is advised to have a sample size exceeding 200 participants for the CFA stage (Marsh et al. 1988; Steenkamp and Maydeu-Olivares 2023). Hence, the number of participants in this study was considered adequate.

### Reliability

To evaluate the reliability, methods assessing internal consistency and stability were employed. The instrument’s overall Cronbach’s alpha, as well as that for each individual item, was calculated to determine internal consistency, with values above .7 being deemed satisfactory (Polit and Beck 2010). Also, test–retest reliability assessment was performed. Fifteen nursing students and five midwifery students who were not among the selected samples completed the questionnaire on 2 separate occasions, 14 days apart.

### Data collection procedure

Data collection was conducted in person, adhering to the student’s educational schedule at the nursing school, and was coordinated with the nursing faculty during classroom sessions. The questionnaires were anonymously completed under the researchers’ guidance within 7–10 minutes, after which they were individually reviewed and sequentially numbered. In the present study, 350 nursing and midwifery students were selected in CFA section, but 14 questionnaires were excluded due to lack of information, resulting in a final participant of 336 students.

### Data analysis

The Waltz & Bausell index (Polit et al. 2007) was used to confirm the quantitative content validity. The EFA and the CFA were used to verify the structure. Skewness and kurtosis were used to examine the normality of data distribution. The Cronbach’s alpha coefficient and test–retest (Gravesande et al. 2019) were used for the tool’s reliability, and the Pearson correlation coefficient was used to determine the internal correlation of the model. All statistical analyses were performed using SPSS 26 and LISREL 8.

## Results

### Descriptive results

A total of 536 participants randomly separated into 2 groups by SPSS statistical software. For EFA, the first group comprised 200 respondents, the second group had 336 respondents for CFA. The demographic and characteristic profiles of the respondents are demonstrated in Table 1.

**Table 1.** Demographic characteristics of participants in study (*N* = 536)

	*N* (%)	
Variables	EFA	CFA
Age	21.65 ± 2.4	21.89 ± 2.22
Gender		
Female	103 (51.5)	197 (58.3)
Male	97 (48.5)	139 (41.7)
Profession		
Nursing St.	165 (82.5)	279 (82.5)
Midwifery St.	35 (17.5)	57 (16.9)
Grade		
First year	32 (16)	54 (16)
Second year	60 (30)	82 (24.3)
Third year	69 (34.5)	128 (37.9)
Fourth year	39 (19.5)	74 (21.9)

### Face validity

In assessing the qualitative face validity, items 14 and 23 was identified as needing revisions to remove any ambiguity. These revisions were made and included in the questionnaire. During the quantitative face validity assessment, all items achieved an impact score greater than 1.5, leading to the retention of all items.

### Content validity

In the qualitative content analysis, 9 experts recommended revisions for 5 specific items (items 5, 9, 13, 18, and 25) to enhance clarity and comprehension. Following the review, the specified items were re-evaluated and subsequently validated by a panel of experts.

The quantitative content validity of the instrument was assessed using the CVR for the entire questionnaire, which was .84 and fell within the range of .57–1. Additionally, the CVI of the instrument, calculated using the Waltz and Bausell index, was .92, with scores ranging from .71 to 1. The skewness of the items ranged from −1.08 to .96, and the kurtosis ranged from −.55 to .92, within the range of (−1, 1), indicating that the items were approximately symmetrical (Table 2).

**Table 2.** The EPICC Spiritual Care Competency Scale – results of CVI, CVR, skewness and kurtosis

No	Items	CVI^a^	CVR^b^	Skewness^c^	Kurtosis^d^
1	I comprehend the notion of spirituality.	.93	.86	−.88	.39
2	I can describe how spirituality affects health and well-being throughout life for myself and others.	.93	.86	−.77	.41
3	I recognize the importance of my values and views while delivering spiritual care.	1.00	1.00	.96	.78
4	I reflect on my values and views, acknowledging that they may differ from others.	1.00	0.71	−.87	.44
5	I care for my well-being.	.93	.86	−1.08	.92
6	I am open to exploring my personal, religious, and spiritual views.	.93	.86	.77	.06
7	I embrace varied spiritual expressions.	.86	.86	−.71	−.041
8	I understand how others express their spirituality.	.71	1.00	−.63	−.071
9	I understand how diverse worldviews and religious beliefs can affect people’s reactions to significant life events.	.86	.71	−.78	.04
10	I acknowledge the individuality of people’s spiritualities.	.93	./57	−.82	.098
11	I communicate with individuals and respond attentively to their spirituality.	1.00	.86	−.59	−.21
12	I am trustworthy, personable, and appreciative of many spiritual expressions and worldviews.	.93	.86	.71	.087
13	I comprehend the idea of spiritual care.	.93	1.00	.55	−.09
14	I am familiar with many approaches to spiritual evaluation.	.93	.86	−.35	−.42
15	I comprehend the responsibilities of other professionals in spiritual care.	.93	.86	−.31	−.45
16	I can analyze and document spiritual needs and resources.	1.00	1.00	−.30	−.33
17	I may partner with other specialists to provide spiritual treatment.	.86	.71	−.56	−.17
18	I can adequately contain and cope with emotions.	.79	.57	−.49	−.55
19	I am honest, friendly, and non-judgmental.	.93	.86	.58	−.26
20	I am willing to cope with emotions.	.93	.86	.57	−.25
21	I comprehend the notion of compassion and presence and their value in spiritual care.	1.00	1.00	.76	.27
22	I comprehend how to respond to recognized spiritual needs and resources effectively.	.93	.86	−.60	−.03
23	I can assess whether the spiritual needs have been met or not.	.93	.86	−.53	−.09
24	I can acknowledge my limitations in providing spiritual care, referring to others as necessary.	1.00	1.00	−0.49	−0.38
25	I analyze and document personal, professional, and organizational components of spiritual care and review as needed.	.86	.71	−.65	−.11
26	I demonstrate compassion and presence.	.79	.57	−.72	.05
27	I am willing to work with and recommend professionals and non-professionals to provide spiritual care.	.93	.86	−.42	−.35
28	I am friendly and receptive, demonstrating empathy, openness, professional humility, and trustworthiness in seeking spiritual help.	.93	.86	−.79	.38
The EPICC Spiritual Care Competency		.92	.84		

a Content validity ratio.

b Content validity index.

c Skewness is a measure of symmetry, or more precisely, the lack of symmetry.

d Kurtosis is a measure of whether the data are heavy-tailed or light-tailed relative to a normal distribution.

### Construct validation results

### Exploratory factor analysis

EFA was performed on 200 initial samples. For EFA, the correlation coefficients of scores between the questionnaire questions were examined and ensured that they were high. The KMO test and Bartlett’s test of sphericity results were .938 and 5748.11, respectively. The KMO value was greater than .9, indicating that the data were excellent for factor analysis. The Sig = .0001 for Bartlett’s test of sphericity justified performing EFA on this questionnaire.

EFA was performed on the responses of the testis and 28 questions of the questionnaire after ensuring the above assumptions. The principal component (PC) analysis method and varimax orthogonal rotation were used to extract factors. In supplementary Table S1, the values of the extracted shares related to each question with the PC method and the results of the stability test pertaining to them are shown.

**Figure 2.**
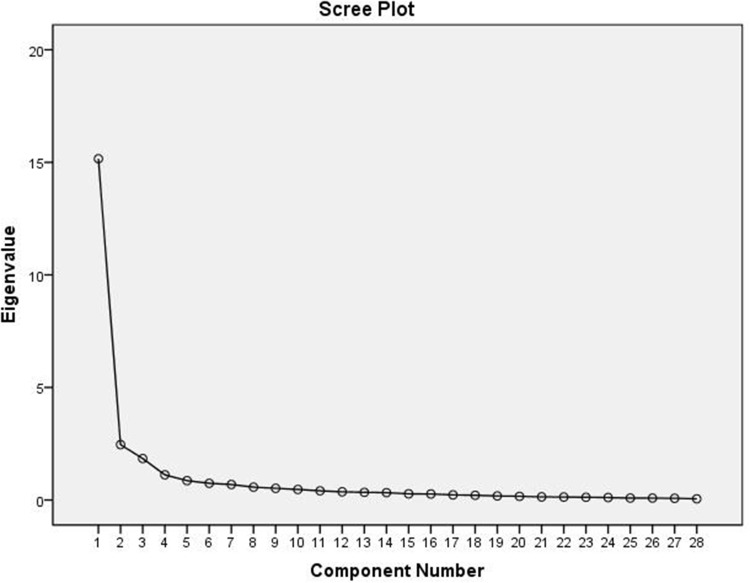
Scree plot of the extracted components of the questionnaire.

Then, the factors that had a percentage of eigenvalues greater than 1 were selected to determine the number of factors. The initial results showed that 4 factors or components could be chosen for analysis. The extracted factors, along with the eigenvalues, the percentage of each factor in explaining the variance of 28 items, and the cumulative variance explained by each of the 4 factors are shown in supplementary Table S2. In total, 4 factors with eigenvalues greater than 1 explained 73.49% of the variance of 28 items. The scree plot from the factor analysis in SPSS software also showed that 4 factors or components were suitable for the final analysis (Figure 2).

Supplementary Table S3 displays the rotated factor matrix. This table identifies the questions that had a factor loading above 0.4 and were allocated to the relevant component. Table 3 shows the extracted factors and their items.

**Table 3.** *T*-value Pearson correlation coefficient and factor loadings of the EPICC Spiritual Care Competency

Factor	No	*t* value^a^	(*λ*)^b^	Cronbach’s Alpha if Item Deleted	
Spiritual care: intervention and evaluation	21	17.45**	.80**	.967	.996
	22	18.43**	.83**	.967	
	23	14.47**	.70**	.967	
	24	16.68**	.78**	.967	
	25	18.51**	.83**	.968	
	26	16.46**	.77**	.968	
	27	15.62**	.74**	.968	
	28	16.93**	.79**	.967	
Spiritual care: assessment and planning	13	11.71**	.61**	.967	.925
	14	12.31**	.63**	.967	
	15	11.2**	.58**	.967	
	16	14.2**	.70**	.967	
	17	13.4**	.67**	.968	
	18	16.8**	.79**	.967	
	19	17.8**	.82**	.968	
	20	16.67**	.78**	.967	
Interpersonal (related to others) spirituality	8	18.48**	.84**	.967	.96
	9	18.61**	.84**	.967	
	10	16.55**	.78**	.967	
	11	17.42**	.81**	.967	
	12	16.94**	.79**	.967	
Intrapersonal (within you) spirituality	1	14.49**	.71**	.967	.893
	2	16.17**	.77**	.967	
	3	16.36**	.77**	.967	
	4	14.41**	.71**	.967	
	5	11.72**	.60**	.967	
	6	13.79**	.68**	.967	
	7	12.93**	.65**	.967	
					.968

** *P* < 0.01.

a The calculated values for all factor loadings of the first and second orders are greater than 1.96 and are therefore significant at the 95% confidence level.

b The specific value, which is denoted by the Lamda coefficient and the statistical symbol λ, is calculated from the sum of the factors of the factor loads related to all the variables of that factor, C. Pearson Correlation coefficient.

### Confirmatory factor analysis

**Figure 3.**
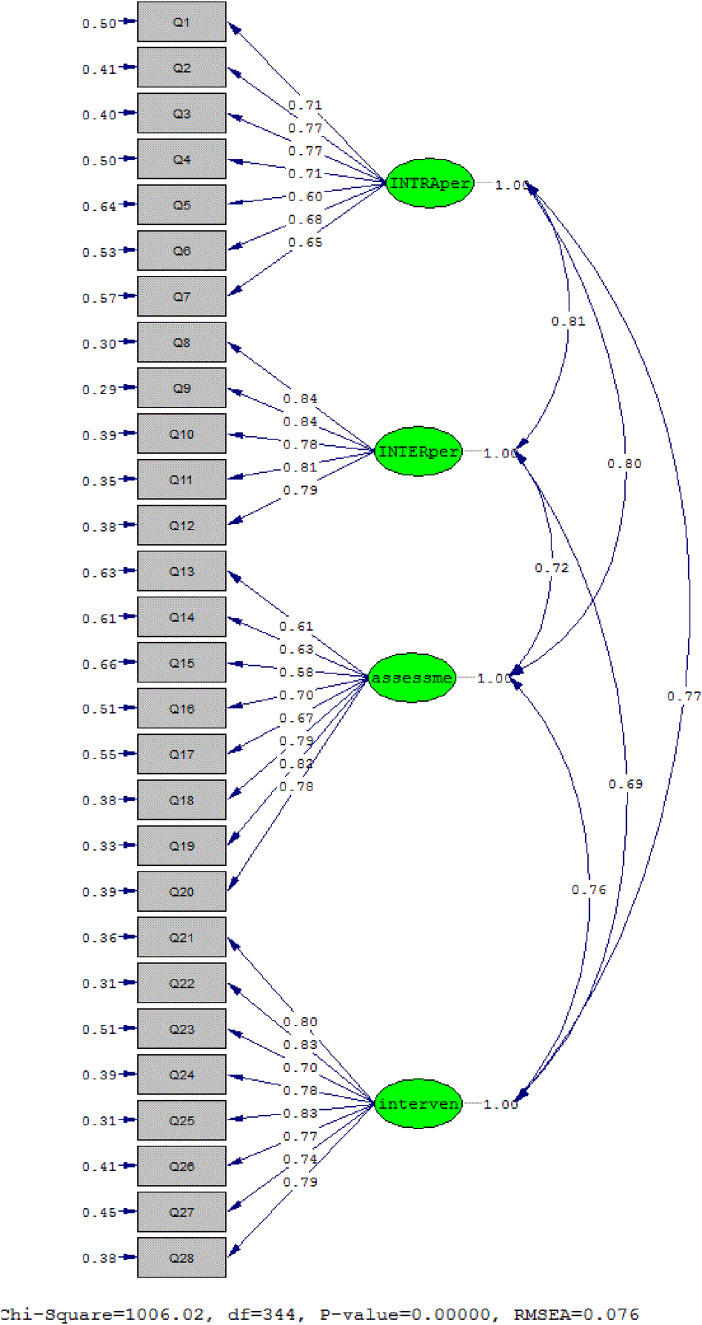
Four-factor model of the EPICC Spiritual Care Competency (Standard).

CFA was performed on 336 samples. The main goal of CFA is to test how well a predefined factor model fits a set of observed data. In other words, CFA checks whether the number of factors and the factor loadings of the measured variables match the theoretical and conceptual model. First, the distribution of data was examined using skewness and kurtosis, which showed that the data were in the range of (−1 and 1) and had a normal distribution. Then, the validity of the model was evaluated by looking at the factor loadings of each question (Table 3). The *t*-values of all items were above 3.29, indicating a significant level of .001. Also, fit indices were used (Table 4) to assess the fit of the model, which showed that the model had a good fit. Therefore, CFA confirmed the questionnaire model with 4 factors and a total of 28 items (Figure 3).

**Table 4.** Fit indices of confirmatory factor analysis model of the EPICC Spiritual Care Competency

Fit indicators	Criterion	Level	Interpretation
*Χ*^2^/*df*	≤3	2.92	Optimal fit
*df*		344	
*X*^2^		1006.02	
*P*_VALUE_		.0001	
NNFI (OR TLI)	>.9	.97	Optimal fit
CFI	>.9	.97	Optimal fit
AGFI	>.8	.91	Optimal fit
SRMR	<.05	.044	Optimal fit
RMSEA	.05–.08	.076	Optimal fit

### Reliability analysis

The internal consistency of the Persian version of the EPICC Spiritual Care Competency was tested using Cronbach’s alpha coefficient of the factors of the scale and test–retest method. The test–retest result was 0.867, indicating a high internal consistency of the Persian version of the EPICC Spiritual Care Competency.

Table 5 demonstrates that Cronbach’s alpha coefficients of all 4 factors of the model were above 0.86, and Cronbach’s alpha coefficient of the whole scale was 0.959.

**Table 5.** Correlation results of the Persian version of the EPICC Spiritual Care Competency and its factors

Factor	Cronbach’s alpha	Intrapersonal spirituality	Interpersonal spirituality	Assessment and planning	Intervention and evaluation	EPICC Spiritual Care Competency
Intrapersonal spirituality	.869	1	.723**	.724**	.70**	.89**
Interpersonal spirituality	.905	.723**	1	.67**	.63**	.84**
Assessment and planning	.884	.724**	.67**	1	.70**	.89**
Intervention and evaluation	.92	.70**	.63**	.70**	1	.88**
EPICC Spiritual Care Competency	.959	.89**	.84**	.89**	.88**	1

** *p*_Value_ < 0.001.

### Correlation between factors

The Pearson correlation test was applied to examine the correlation between the factors of the Persian version of the EPICC Spiritual Care Competency (the data of the main scale and the subscales had a normal distribution based on the Kolmogorov–Smirnov test). Table 5 indicates that there was a significant relationship between all factors and the main scale at the 99% level (*P* < .01). There was also a significant correlation between the factors of the scale at the 99% confidence level (*P* < .01). Therefore, the scale and its factors had a high correlation.

## Discussion

The present study showed that the EPICC questionnaire had adequate validity and reliability among Iranian nursing and midwifery students. The KMO value in this study was .938, which was close to the value of .9 in Giske et al.’s study (Giske et al. 2023) and the value of 0.86 in Fopka-Kowalczyk et al.’s study. Fopka-Kowalczyk et al. developed and psychometrically tested the Spiritual Care Support Tool as a new tool for assessing spiritual care competency (Fopka-Kowalczyk et al. 2023) The PC method and the varimax orthogonal rotation were used to extract the factors in the factor analysis and identify 4 factors. Giske et al. identified 5 factors in their study. Most of the items in Giske et al.’s study loaded on the first factor (Giske et al. 2023). However, in this study, only the third factor had 5 items, and the other factors had 7 or 8 items with eigenvalues above 0.4. Fopka-Kowalczyk et al. identified 5 factors for 31 items of the Spiritual Care Support Tool using the oblimin rotation (Fopka-Kowalczyk et al. 2023). In terms of the results, the method of analyzing the study, selecting the rotation type in EFA, the sample size, and how participants answered the scale can impact the differences between this study’s results and those of other studies. Additionally, students’ attitudes toward spiritual care, the influence of religious perspectives, and how subjects perceive meanings and concepts can also vary across different societies.

Hu et al. psychometrically tested the Spiritual Care Competency Scale in Palliative Care, which is a self-report scale, in China in 2019. Their study showed that the tool (PCSCCS-M) had a KMO value of .936. They extracted 3 factors from this questionnaire, which measured 1 – knowledge and skill, 2 – self-awareness and attitude, and 3 – spiritual care that meets the spiritual needs of the patient (Hu et al. 2019).The 4 factors extracted in this study also measured the knowledge and skill in performing spiritual care, similar to Giske et al.’s study (Giske et al. 2023), which was consistent with the cited studies.

The CFA confirmed the questionnaire model with 4 factors and a total of 28 items. All factors also had a significant and appropriate correlation with each other. However, in Giske et al.’s study, the CFA showed that 2 items loaded on factor 4 had a low correlation with other subscales (Giske et al. 2023). This study was not consistent with Giske et al.’s study in this regard. In this regard, we can say that the structure and of the dominant culture in Iranian society differ and share similarities with other countries. Cultural variations, attitudes, and concepts related to the religious dimension of spirituality play a significant role. The discussion of spiritual care, a comprehensive concept influenced by culture, religion, religious perspectives, and attitudes toward caregiving, is essential (Murgia et al. 2022).

The Cronbach’s alpha coefficient (.968) indicated the adequate reliability of the tool. The Cronbach’s alpha coefficient in this study was higher than the Cronbach’s alpha coefficient in Giske et al.’s study (.91) (Giske et al. 2023) and Fopka-Kowalczyk et al.’s study (.88) (Fopka-Kowalczyk et al. 2023). The correlation between the items in this study ranged from .6 to .7, which was higher than Giske et al.’s study and Fopka-Kowalczyk et al.’s study. The literature review suggested that the minimum acceptable level for the correlation between the items was .15 (Mamier and Taylor 2015). Therefore, the item-total correlation was also adequate. In this regard, it can be argued that religious and cultural reasons may make spiritual care more frequent in religious and Muslim countries such as Iran (Taylor et al. 2023).

As the literature review showed, different tools for assessing spiritual care competency have similar validity and reliability across other communities with diverse cultures and religions. The meaning of spiritual care accounts for these similarities. Spirituality is a basic human need that connects to the meaning and purpose of life (Merati-Fashi et al. 2021).

## Limitations

The present study had some limitations that should be considered. The sample size was adequate for the study, but the ratio of midwifery students to nursing students was low. In this study, we had to choose universities from the western regions of Iran because of the limited number of students’ available and specific criteria. We selected nursing and midwifery students from various educational levels. Specifically, we chose students with a minimum of 1 semester of academic background and practical patient care experience during their internship. It’s suggested to conduct larger studies with more nursing and midwifery students. These studies would include different methods to ensure reliability and validate results.

## Conclusion

The findings indicated that the indigenized EPICC Spiritual Care Competency tool in Iran has adequate validity and reliability and can be used by health-care providers in clinical settings. Considering the nature and essence of the nursing and midwifery profession, which requires prolonged interactions with patients, the students employed in this field can provide better care for the patients if they have higher spiritual health. Since the education system in Iran is centralized and consistent throughout the country, nursing and midwifery instructors and planners can use the findings of this study to improve the quality and revise the curriculum of spiritual care for students.

## Supporting information

Jalali et al. supplementary material Jalali et al. supplementary material

## Data Availability

The datasets supporting the conclusions of this study are available upon reasonable request from the corresponding author.

## Supplementary material

The supplementary material for this article can be found at https://doi.org/10.1017/S147895152400141X.
