# Supplementary material for: Assessment of psychometric properties of the Persian version of the spiritual care competency self-assessment tool
Source: Palliat Support Care. 2025 Jan 21;23:e37. doi: 10.1017/S147895152400141X (PMC13166569; doi:10.1017/S147895152400141X)
Supplement: Jalali et al. supplementary material [file S147895152400141Xsup001.docx]

**Supplementary table 1.** Extracted eigenvalues for each sol and stability test

| No | Initial | Extraction | Corrected Item-Total Correlation | Cronbach's Alpha if Item Deleted |
| --- | --- | --- | --- | --- |
| Q1 | 1.000 | .688 | .695 | .967 |
| Q2 | 1.000 | .688 | .739 | .967 |
| Q3 | 1.000 | .749 | .739 | .967 |
| Q4 | 1.000 | .617 | .691 | .967 |
| Q5 | 1.000 | .503 | .560 | .968 |
| Q6 | 1.000 | .632 | .649 | .968 |
| Q7 | 1.000 | .463 | .611 | .968 |
| Q8 | 1.000 | .837 | .699 | .967 |
| Q9 | 1.000 | .866 | .748 | .967 |
| Q10 | 1.000 | .834 | .735 | .967 |
| Q11 | 1.000 | .903 | .751 | .967 |
| Q12 | 1.000 | .852 | .745 | .967 |
| Q13 | 1.000 | .527 | .650 | .968 |
| Q14 | 1.000 | .751 | .703 | .967 |
| Q15 | 1.000 | .514 | .657 | .968 |
| Q16 | 1.000 | .617 | .712 | .967 |
| Q17 | 1.000 | .595 | .680 | .967 |
| Q18 | 1.000 | .820 | .687 | .967 |
| Q19 | 1.000 | .821 | .708 | .967 |
| Q20 | 1.000 | .786 | .709 | .967 |
| Q21 | 1.000 | .848 | .747 | .967 |
| Q22 | 1.000 | .862 | .772 | .967 |
| Q23 | 1.000 | .794 | .751 | .967 |
| Q24 | 1.000 | .761 | .742 | .967 |
| Q25 | 1.000 | .826 | .773 | .967 |
| Q26 | 1.000 | .787 | .754 | .967 |
| Q27 | 1.000 | .798 | .721 | .967 |
| Q28 | 1.000 | .840 | .770 | .967 |
| Extraction Method: Principal Component Analysis. | | |  |  |

**Supplementary table 2**: Variance percentage and specific values of different factors

| Component | Initial Eigenvalues | | | Extraction Sums of Squared Loadings | | | Rotation Sums of Squared Loadings | | |
| --- | --- | --- | --- | --- | --- | --- | --- | --- | --- |
|  | Total | % of Variance | Cumulative % | Total | % of Variance | Cumulative % | Total | % of Variance | Cumulative % |
| 1 | 15.158 | 54.135 | 54.135 | 15.158 | 54.135 | 54.135 | 6.513 | 23.262 | 23.262 |
| 2 | 2.460 | 8.785 | 62.920 | 2.460 | 8.785 | 62.920 | 5.115 | 18.269 | 41.531 |
| 3 | 1.842 | 6.577 | 69.497 | 1.842 | 6.577 | 69.497 | 4.704 | 16.801 | 58.332 |
| 4 | 1.119 | 3.996 | 73.494 | 1.119 | 3.996 | 73.494 | 4.245 | 15.162 | 73.494 |

**Supplementary table3**: Matrix of factor loadings of the EPICC Spiritual Care Competency items on components after rotatio

| No | Component | | | |
| --- | --- | --- | --- | --- |
|  | 1 | 2 | 3 | 4 |
| Q1 | .258 | .250 | .264 | .700 |
| Q2 | .249 | .373 | .297 | .631 |
| Q3 | .249 | .238 | .371 | .702 |
| Q4 | .251 | .256 | .345 | .608 |
| Q5 | .309 | .117 | .150 | .610 |
| Q6 | .176 | .266 | .265 | .678 |
| Q7 | .271 | .324 | .189 | .498 |
| Q8 | .192 | .235 | .827 | .248 |
| Q9 | .283 | .213 | .817 | .271 |
| Q10 | .240 | .220 | .795 | .309 |
| Q11 | .232 | .232 | .843 | .291 |
| Q12 | .234 | .241 | .803 | .307 |
| Q13 | .217 | .534 | .248 | .365 |
| Q14 | .241 | .771 | .177 | .261 |
| Q15 | .239 | .500 | .280 | .358 |
| Q16 | .328 | .585 | .358 | .196 |
| Q17 | .197 | .593 | .359 | .275 |
| Q18 | .245 | .841 | .160 | .163 |
| Q19 | .267 | .825 | .130 | .226 |
| Q20 | .301 | .795 | .160 | .193 |
| Q21 | .847 | .214 | .177 | .229 |
| Q22 | .843 | .228 | .215 | .231 |
| Q23 | .795 | .280 | .226 | .178 |
| Q24 | .771 | .298 | .206 | .189 |
| Q25 | .809 | .241 | .241 | .235 |
| Q26 | .782 | .293 | .152 | .258 |
| Q27 | .822 | .256 | .148 | .189 |
| Q28 | .806 | .150 | .227 | .341 |
| Extraction Method: Principal Component Analysis.  Rotation Method: Varimax with Kaiser Normalization. | | | | |
| a. Rotation converged in 6 iterations. | | | | |
